# Effect of Extracellular Matrix Stiffness on Candesartan Efficacy in Anti-Fibrosis and Antioxidation

**DOI:** 10.3390/antiox12030679

**Published:** 2023-03-09

**Authors:** Tong Zhu, Jingjing Song, Bin Gao, Junjie Zhang, Yabei Li, Zhaoyang Ye, Yuxiang Zhao, Xiaogang Guo, Feng Xu, Fei Li

**Affiliations:** 1 The Key Laboratory of Biomedical Information Engineering of Ministry of Education, School of Life Science and Technology, Xi’an Jiaotong University, Xi’an 710049, China; tongzhu@stu.xjtu.edu.cn (T.Z.); jingjingsong@stu.xjtu.edu.cn (J.S.); junjie-zhang@stu.xjtu.edu.cn (J.Z.); yzy3119113038@stu.xjtu.edu.cn (Z.Y.); yuxiangzhao@stu.xjtu.edu.cn (Y.Z.); fengxu@mail.xjtu.edu.cn (F.X.); 2 Bioinspired Engineering and Biomechanics Center (BEBC), Xi’an Jiaotong University, Xi’an 710049, China; liyabei@stu.xjtu.edu.cn; 3 Department of Cardiovasology, Xidian Group Hospital, Xi’an 710077, China; 4 Department of Endocrinology, Tangdu Hospital, Air Force Military Medical University, Xi’an 710032, China; bingao@fmmu.edu.cn; 5 School of Chemistry, Xi’an Jiaotong University, Xi’an 710049, China; 6 Department of Cardiology, The First Affiliated Hospital, Zhejiang University School of Medicine, Hangzhou 310003, China; gxg22222@zju.edu.cn

**Keywords:** myocardial fibrosis, candesartan (CAN), extracellular matrix (ECM), oxidative stress, scanning electrochemical microscopy (SECM)

## Abstract

Myocardial fibrosis progression and imbalanced redox state are closely associated with increased extracellular matrix (ECM) stiffness. Candesartan (CAN), an angiotensin II (Ang II) receptor inhibitor, has shown promising anti-fibrosis and antioxidant efficacy in previous cardiovascular disease studies. However, the effect of ECM stiffness on CAN efficacy remains elusive. In this study, we constructed rat models with three different degrees of myocardial fibrosis and treated them with CAN, and then characterized the stiffness, cardiac function, and NADPH oxidase-2 (NOX2) expression of the myocardial tissues. Based on the obtained stiffness of myocardial tissues, we used polyacrylamide (PA) gels with three different stiffness to mimic the ECM stiffness of cardiac fibroblasts (CFs) at the early, middle, and late stages of myocardial fibrosis as the cell culture substrates and then constructed CFs mechanical microenvironment models. We studied the effects of PA gel stiffness on the migration, proliferation, and activation of CFs without and with CAN treatment, and characterized the reactive oxygen species (ROS) and glutathione (GSH) levels of CFs using fluorometry and scanning electrochemical microscopy (SECM). We found that CAN has the best amelioration efficacy in the cardiac function and NOX2 levels in rats with medium-stiffness myocardial tissue, and the most obvious anti-fibrosis and antioxidant efficacy in CFs on the medium-stiffness PA gels. Our work proves the effect of ECM stiffness on CAN efficacy in myocardial anti-fibrosis and antioxidants for the first time, and the results demonstrate that the effect of ECM stiffness on drug efficacy should also be considered in the treatment of cardiovascular diseases.

## 1. Introduction

Myocardial fibrosis, a common pathological process that occurs during the progression of various cardiovascular diseases, exhibits an imbalance between the production and degradation of extracellular matrix (ECM), which results in an increase in ECM stiffness [1,2], and the persistently high ECM in turn augments myocardial fibrosis progression [3,4]. For instance, ECM stiffness participates in the production of angiotensin II (Ang II) in cardiomyocytes, affecting the secretion of collagen in cardiac fibroblasts (CFs) [5] and regulating CF cytoskeleton through integrin and promoting their differentiation [6]. Moreover, ECM stiffness induces cellular oxidative stress by increasing the activity of nicotinamide adenine dinucleotide phosphate (NADPH) oxidase (NOX), leading to imbalanced cellular glutathione (GSH) [7] and reactive oxygen species (ROS) [8]. Thus, considering the key role of ECM stiffness in myocardial fibrosis development, it is important to research the effect of ECM stiffness on the physiological functions of CFs.

Mechanical and biochemical factors are interdependent in the biological processes of CFs differentiation [9]. For instance, mechanical signals of cardiac cell membrane tension induced by ECM stiffness can be transmitted to cells through integrin [10], and cardiac cells respond to ECM stiffness through mechanical signal transduction molecules on the cell membrane. AT_1_R, a key membrane protein in the renin-angiotensin system (RAS), is considered an important receptor for mechanical signaling [11], and its activation can lead to local contact formation and cell contraction. In addition, it has been reported that ECM stiffness can directly induce the up-regulation of AT_1_R expression without the involvement of angiotensin, indicating that AT_1_R also has the mechanical signal transduction effect [5]. Focal adhesion kinase (FAK), a widely expressed tyrosine kinase and a downstream component of an integrin-regulated signaling pathway, participates in the transmission of mechanical signals [12,13]. FAK phosphorylation and its consequent activation regulate several basic biological functions of cells, such as cell migration [14] proliferation [15], and adhesion [16], and are involved in cardiomyocyte hypertrophy [17] and cellular oxidative stress [18]. Thus, it is reasonable to speculate that AT_1_R and FAK may be involved in the process that ECM stiffness affects the progression of myocardial fibrosis.

Mechanical properties of ECM not only regulate the biochemical behavior of cells but also affect the pharmacodynamics and therapeutic efficacy of drugs. For example, previous studies reported that increased bronchial wall stiffness reduced bronchodilator-induced bronchodilation [19], and increased ECM stiffness aggravated the multidrug resistance of tumors [20]. For cardiovascular diseases, previous reports mainly focused on the response of dynamic mechanics (e.g., blood shear force or tensile force) to the therapeutic effect of cardiovascular drugs, while understanding the effect of static ECM stiffness on the cardiovascular drugs efficacy in myocardial fibrosis is still limited. Candesartan (CAN), a classic angiotensin II (Ang II) receptor inhibitor, has been widely used as a first-line drug for treatments of coronary heart disease, heart failure, hypertension, and other cardiovascular diseases in clinic [21]. Many clinical studies have proven that CAN can provide prevention and regression of left ventricular hypertrophy and cardiac fibrosis, protect heart against ischemia-reperfusion injury and reduce myocardial damage during myocarditis [22,23], and improve oxidative stress state of heart [24,25]. Thus, it is essential to clarify the ECM stiffness effect on the efficacy of CAN.

In this work, we first obtained the cardiac tissue stiffness by constructing rat models with three different degrees of myocardial fibrosis and treated them with/without CAN. Then, we cultured CFs with/without CAN intervention on the PA gels with different stiffness to mimic the ECM stiffness of CFs at three degrees of myocardial fibrosis. The therapeutic effect of CAN was evaluated by comparing the myocardial fibrosis index and oxidative stress before and after CAN treatment both in vivo and in vitro. Finally, based on the p-FAK and AT1R expression results, we discussed the possible mechanism of the ECM stiffness effect on the efficacy of CAN in myocardial fibrosis. Our study proves the interaction between ECM stiffness and CAN efficacy in myocardial fibrosis, which can provide useful insights into the anti-fibrosis and antioxidant efficacy of CAN from the biomechanopharmacology perspective.

## 2. Materials and Methods

For any procedures not mentioned below, see supplementary data online.

### 2.1. Animals

Male Sprague-Dawley rats (6–8 weeks old and weighing 250–300 g) were obtained from the experimental animal center of the School of Medicine of Xi’an Jiaotong University. The myocardial fibrosis model was constructed by subcutaneous injection of isoprenaline (ISO, 5 mg/kg/day, Sigma-Aldrich, St. Louis, MO, USA) once daily for 7–28 days. The treatment groups of rats received CAN (2 mg/kg/day, Sigma-Aldrich, St. Louis, MO, USA) by gastric gavage once daily for 28 days. The detailed treatment procedure of rats is presented in Table 1 and the rats were randomly divided into six groups. All experimental protocols were approved by the Biomedical Ethics Committee of the Medicine Department of Xi’an Jiaotong University (approval number: 2022-1515). In vivo experiments and animal management procedures were carried out according to the NIH Guide for Care and Use of Laboratory Animals [26].

### 2.2. Assessment of Cardiac Functions of Rats

The cardiac function of rats was assessed using an ultrasonic diagnostics instrument (IE33, Philips, Amsterdam, The Netherlands) equipped with an S12-4 linear array ultrasound transducer.

### 2.3. Staining Masson’s Trichrome

Paraffin sections were stained with a Masson’s trichrome staining kit (Servicebio, Wuhan, China). The ratios of the stained fibrotic areas to the total ventricular areas were calculated and used as the collagen volume fraction.

### 2.4. Measurement of Young’s Modulus of Myocardial Tissue

**Figure 1:**
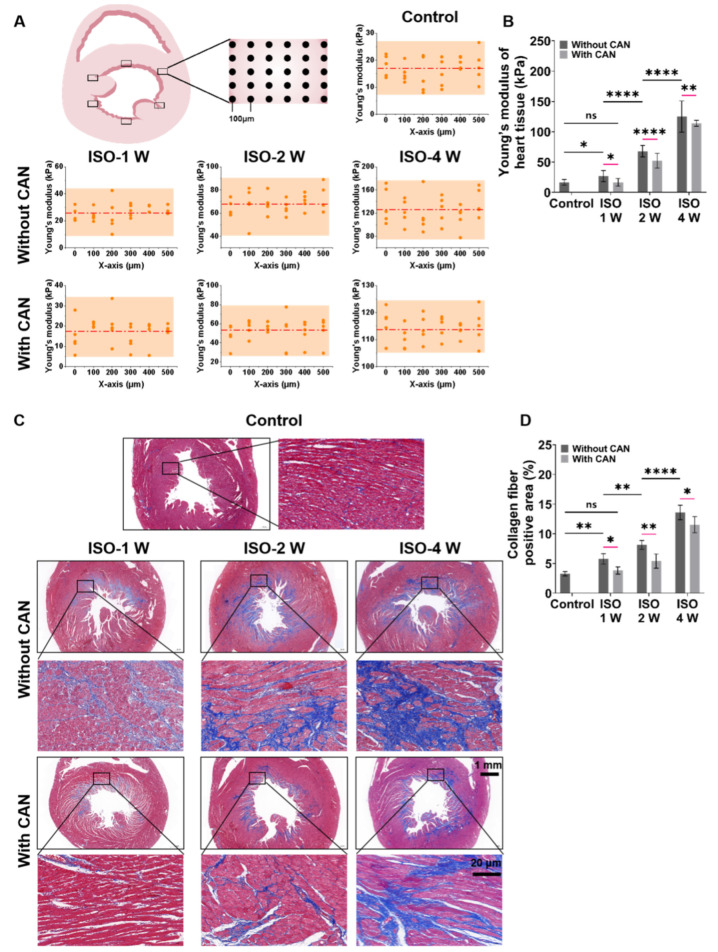
Characterization results of myocardial tissue stiffness and collagen deposition in myocardial fibrosis rats without and with CAN treatment. (**A**) Stiffness diagrams of tissue samples were obtained based on their Young’s moduli measured by nanoindentation instrument. (**B**) Statistical histograms of tissue stiffness of fibrotic myocardium (*n* = 5). (**C**) Representative Masson’s trichrome staining images of fibrotic myocardium without and with CAN treatment. (**D**) Statistical histograms of collagen fiber-positive area (%) (*n* = 5). Data are shown as means ± SEM. ns, no significant difference, * *p* < 0.05, ** *p* < 0.01 and **** *p* < 0.0001 determined by two-way ANOVA.

The myocardial tissues of rats were sliced into sections of approximately 500-μm thickness and glued on glass slides followed by immersion in phosphate-buffered saline (PBS). The stiffness of the slices (Young’s modulus, *E*) was measured using a Nanoindenter instrument (Piuma, Optics11, Amsterdam, The Netherlands) with a probe with a radius of 47 µm and a cantilever stiffness of 0.5 N/m. We randomly selected six locations on the whole ventricular wall in the tissue slice for measurements and indented the probe at each position 30 times (5 × 6 matrix) to measure the stiffness (Schematic diagram in Figure 1A).

### 2.5. Measurement of Brain Natriuretic Peptide (BNP) and Cardiac Troponin T (c-TnT)

Blood samples of rats were collected from the abdominal aorta and centrifuged for 15 min at 4000 rpm to obtain the plasma. The contents of brain natriuretic peptide (BNP) and cardiac troponin T (c-TnT) in plasma were measured using a commercial enzyme-linked immunosorbent assay kit (Solarbio, Beijing, China) according to the manufacturer’s instructions. The absorbance of the plasma sample at 450 nm was recorded using a Spark 10 M Multimode Microplate Reader (TECAN, Männedorf, Switzerland).

### 2.6. Immunohistochemical Analysis of AT_1_R, p-FAK, FAK and NOX2

Paraffin sections of rat heart were incubated overnight with the anti-angiotensin II type-1 receptor antibody (AT_1_R, 1:100 dilution, Proteintech, 25343-1-AP, Chicago, IL, USA), the anti-focal adhesion kinase (phospho Y397) antibody (p-FAK, 1:800 dilution, Abcam, ab81298, Cambridge, UK), anti-focal adhesion kinase antibody (FAK, 1:800 dilution, Abcam, ab40794, Cambridge, UK) and NOX2 rabbit polyclonal antibody (1:400 dilution, Servicebio, Wuhan, China) at 4 °C and subsequently washed with PBS for three times. Then, the sections were incubated with second antibodies includes: IgG-horseradish peroxidase (HRP) (1:100; Dako, Wuhan, China; P0448, Copenhagen, Denmark) and Alexa Fluor-488 goat anti-rabbit antibody (1:500 dilution, Servicebio, Wuhan, China) at room temperature for 1 h. Finally, the sections were incubated with 4′,6-diamidino-2-phenylindole (DAPI). After dying, 3,3′ diaminobenzidine tetrahydrochloride (DAB) horseradish peroxidase Color Development Kit (Hat Biotechnology, Wuhan, China; IS015) was used for chromogenic development. Microphotographs were acquired and analyzed with fluorescence microscopy (ECLIPSE C1, Nikon, Tokyo, Japan). The analysis of the fluorescence area was performed using CaseViewer2.4 software.

### 2.7. Preparation and Characterization of Polyacrylamide (PA) Gels

PA gels for culturing CFs were prepared using the procedure described in the previous literature [27,28]. First, a precursor solution containing 40% (*w*/*v*) acrylamide monomers (Macklin, Shanghai, China), 2% (*w*/*v*) *N*,*N*-methylene-bis-acrylamide (MBA) (Macklin), 10% (*w*/*v*) ammonium persulfate (APS, Sigma-Aldrich) and tetramethylethylenediamine (TEMED, Sigma-Aldrich) were prepared. To prepare the PA gels with different stiffness (29.4 kPa, 67.7 kPa, and 125.5 kPa), the mass/volume concentrations of APS and TEMED were kept at 1% and 0.1% and the ratios of acrylamide (%)/MBA (%) were 10/0.3, 10/0.5 and 15/0.9, respectively. Then, 50 μL of the prepared polymer solution was dropped onto the hydrophilic-treated glass bottom of a petri dish, and an 18 mm-in-diameter glass coverslip treated with dichlorodimethylsilane was carefully placed on the top of the solution. After the PA gel polymerization, the top coverslips were peeled off, and the remaining monomers and cross-linkers were removed by washing with PBS. Then, 1 mg mL^−1^ of the cross-linker N-sulfosuccinimidyl 6-(4′-azido-2′-nitrophenyl amino) hexanoate (Sulfo SANPAH, Thermo Scientific, Waltham, MA, USA) was added to the PA gels and photoactivated through ultraviolet light exposure for 10 min. The PA gels were then washed with 50 mM HEPES (pH 8.5) and incubated overnight in a solution of 50 μg mL^−1^ rat tail tendon collagen type I (Corning, Corning, NY, USA). The Young’s modulus of the PA gels was measured by the nanoindenter instrument (Piuma, Optics11, Amsterdam, The Netherlands).

### 2.8. Isolation and Purified Culture of Neonatal Rat Cardiac Fibroblasts

Neonatal rat cardiac fibroblasts (NRCFs) were isolated from 1–3-day-old Sprague-Dawley rats following the NIH guidelines [26]. In Brief, chopped myocardial tissues were dispersed in 2 mg mL^−1^ collagenase type II enzyme solution at 37 °C for vibration digestion several times. The dissociated cells were suspended in DMEM/F12 (Corning, Corning, NY, USA). Then, the cells were obtained by filtering and centrifuging the mixed solution. The differential adhesion method was used to obtain NRCFs after 1 h. The purity of NRCFs was determined by vimentin protein staining and the NRCFs were extracted before each experiment.

### 2.9. Immunofluorescence Staining of α-Smooth Muscle Actin, p-FAK and AT_1_R

CFs were penetrated and incubated with the following primary antibodies at 4 °C overnight: anti-alpha smooth muscle actin antibody (α-SMA, 1:800 dilution, Abcam, ab7817, Cambridge, UK), anti-focal adhesion kinase (phospho Y397) antibody (FAK, 1:800 dilution, Abcam, ab81298, Cambridge, UK), and anti-angiotensin II type-1 receptor antibody (AT_1_R, 1:100 dilution, Proteintech, 25343-1-AP, Chicago, IL, USA). Then, the cell samples were incubated with the following secondary antibodies in the dark at 37 °C for 2 h: Alexa Fluor-488 goat anti-mouse antibody (1:1000 dilution, Abcam, Ab150077, Cambridge, UK) or Alexa Fluor-594 goat anti-rabbit antibody (1:1000 dilution, Abcam, Ab150116, Cambridge, UK). Cell nuclei were stained using DAPI (1 μg mL^−1^, Sigma, D9542, St. Louis, MO, USA). Images of the cell samples were obtained using a laser scanning confocal microscope (FV3000 Olympus, Tokyo, Japan).

### 2.10. Western Blotting

The expression levels of α-SMA, p-FAK, AT_1_R, collagen I (COL I, 1:1000 dilution, Abcam, ab270993, Cambridge, UK), collagen III (COL III, 1:1000 dilution, Sigma, c7805, St. Louis, MO, USA), and matrix metalloproteinase-2 (MMP-2, 1:1000 dilution, Abcam, ab92536, Cambridge, UK) were determined by western blotting (WB). All cell samples were lysed in RIPA lysis buffer (Solarbio, Beijing, China). The protein concentration was determined using a bicinchoninic acid (BCA) protein assay kit (Beyotime, Shanghai, China). The protein samples were loaded into the prepared SDS-PAGE separation gel (8% (*w*/*v*) acrylamide gel) and concentrated gel (5% (*w*/*v*) acrylamide gel), followed by electrophoresis under a constant voltage of 80 V. When the protein samples ran to the interface between the concentrated gel and the separated gel, the electrophoresis was converted to 120 V constant voltage until the marker ran to the bottom of the separated gel. Then, the target proteins are transferred to polyvinylidene difluoride (PVDF) membranes (Millipore Bedford, MA, USA) by electrophoresis under a constant current of 230 mA. After transferring the membrane, the PVDF membrane was immersed in the configured skim milk (5%) for sealing. After sealing, the diluted primary antibodies were added to the PVDF membrane and incubated at room temperature for 2 h. After the primary antibody was well incubated, the secondary antibody was added and incubated at room temperature for 2 h. The immunoreactive bands were obtained using a chemiluminescence imaging system (ChemiQ 4800 mini, Ouxiang, Shanghai, China). The results were quantified using ImageJ software and normalized to those of GAPDH.

### 2.11. SECM Measurements of Extracellular GSH Levels

Before SECM measurements, the CFs were incubated in an L15 culture medium (Solarbio, Beijing, China) containing 0.5 mM ferrocenecarboxylic acid (FcCOOH, Aladdin, Shanghai, China) for 30 min. All the SECM measurements were conducted using a commercial SECM instrument (ElProScan3, HEKA Elektronik GmbH, Harvard Bioscience Inc., USA) integrated with an inverted optical microscope (Olympus-IX53, Olympus Co., Ltd., Tokyo, Japan), and a typical three-electrode system with a 10 μm-in-diameter Pt disk electrode (i.e., Pt microelectrode) as the SECM probe and the working electrode, a platinum wire (0.5 mm in diameter) as the counter electrode and an Ag/AgCl wire (0.6 mm in diameter) as the reference electrode.

The highest point of a single CF was determined using a line scanning method along the *x*-axis and *y*-axis on the cell surface with aid of an inverted optical microscope [7,29]. The Pt microelectrode was placed approximately 20 μm above the highest point of the CF. Subsequently, the probe biased at 0.5 V controllably approached the cell surface in the *z*-axis direction with a speed of 0.5 μm s^−1^ with recording the approach curve, from which the *z*-axis position of the highest point of CF was determined. For the cell length characterization, the longest distance in the *x*-axis or *y*-axis was selected as the length of CF. For the cell height characterization, the approach curves to the highest point of CF, and the surface of the PA gel next to the CF were recorded. The height of the CF was obtained by the difference in the absolute distances of the two approach curves. The obtained average heights and lengths of the CFs on the PA gels were inputted into the SECM simulation model (the specific data and description are provided in the Supplementary Materials).

To quantitatively analyze the content of the GSH released by CFs on the PA gels, we used COMSOL multiphysics software (COMSOL Inc., Sweden) with finite element simulation to acquire the regeneration rate (*k*) of FcCOOH, which represent the outflow rates of GSH. The model geometry, simulation parameters, and boundary conditions of the developed simulation model are described in detail in the Supplementary Materials.

### 2.12. Statistical Analysis

Statistical analysis was performed using GraphPad Prism 9 (GraphPad Software, La Jolla, CA, USA). Statistics are presented as the mean ± standard error of the mean (SEM) for all quantitative data, with *n* = 5 for the animal experiments and *n* ≥ 3 for the cell experiments. Statistical significance was evaluated using two-way ANOVA followed by pair comparison with the Tukey test (ns, no significant difference, * *p* < 0.05, ** *p* < 0.01, *** *p* < 0.001, and **** *p* < 0.0001).

## 3. Results

### 3.1. CAN Treatment Results in Decreased Myocardial Stiffness and Collagen Content in Myocardial Fibrosis Rats

The rat models with three degrees of myocardial fibrosis were established through subcutaneous injection of ISO for different days in rats. The heart weight index of the three groups of rats without and with CAN treatment has no significant difference (Supplemental Figure S1). After one, two, and four weeks of ISO injections, the measured Young’s moduli of the myocardial tissues are 26.4 ± 6.1, 67.9 ± 9.3, and 125.1 ± 25.3 kPa, respectively. After treatment with CAN, the Young’s moduli of the myocardial tissues are 17.6 ± 6.1, 52.1 ± 12.1, and 114.0 ± 4.8 kPa, respectively (Figure 1A). These results indicate that CAN can restrain the stiffening of fibrotic myocardium as induced by ISO, and the stiffness of myocardial tissue in the ISO-2 W group decreases most among the three groups with CAN treatment (Figure 1B). Since the increased stiffness of myocardial tissue has been proven to be mainly related to collagen deposition, we measured the collagen contents. From Masson’s trichrome staining results (Figure 1C), we can see that more collagen deposition forms in the myocardial tissue by prolonging the ISO induction time. With CAN treatment, the absolute contents of collagen deposition in myocardial tissues decrease by 2.40%, 2.73%, and 1.97% in the groups of ISO-1 W, ISO-2 W, and ISO-4 W, respectively (Figure 1D). The decrease of collagen deposition in the ISO-2 W group is substantially higher than those of the ISO-1 W and ISO-4 W groups. That is, compared with the groups without CAN treatment, the ISO-2 W group has the most significant decrease in tissue stiffness and collagen content than those in the ISO-1 W and ISO-4 W groups.

### 3.2. CAN Treatment Improves the Cardiac Function of Myocardial Fibrosis Rats

**Figure 2:**
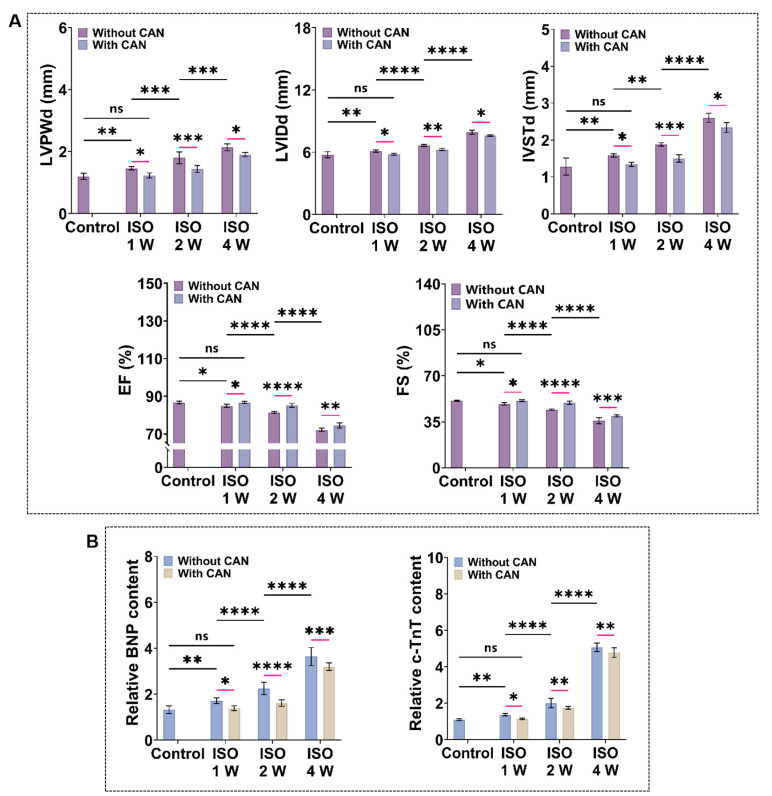
Characterization results of cardiac function and myocardial injury markers in myocardial fibrosis rats without and with CAN treatment. (**A**) Statistical histogram of echocardiographic statistics of myocardial fibrosis rats without and with CAN treatment (*n* = 5). (**B**) Statistical histogram of relative contents of BNP and c-TnT in plasma of myocardial fibrosis rats without and with CAN treatment (*n* = 5). Data are shown as means ± SEM. ns, no significant difference, * *p* < 0.05, ** *p* < 0.01, *** *p* < 0.001, and **** *p* < 0.0001 determined by two-way ANOVA.

To quantify the cardiac function of myocardial fibrosis rats, we measured the echocardiography and plasma markers of heart failure and myocardial injury (BNP and c-TnT). From the ultrasonic indicators (Figure 2A), we observed that the ventricular cavity of myocardial fibrosis rats gradually increases, and the ventricular wall becomes thickened, while the cardiac systolic function gradually decreases with the prolonged ISO induction time. Additionally, the levels of BNP and c-TnT also increase with the extension of ISO induction time (Figure 2B). The ultrasound data and plasma marker levels of the rats treated with CAN are all ameliorated. The average values of LVIDd decreased by 0.16 ± 0.020, 0.38 ± 0.007, and 0.26 ± 0.012 mm, and the average values of IVSTd decreased by 0.04 ± 0.020, 0.38 ± 0.065 and 0.14 ± 0.038 mm, while the average values of LVPWd decrease by 0.18 ± 0.008, 0.46 ± 0.049 and 0.45 ± 0.010 mm. In contrast, the average values of EF and FS increase by 0.56% and 0.6% in the ISO-1 W group, 3.42% and 5.3% in the ISO-2 W group, and 3.03% and 5.07% in the ISO-4 W group. The improvements in the cardiac structure and function in the ISO-2 W group are the best among the three groups under CAN treatment. Furthermore, the decrease of the myocardial injury indexes in the ISO-2 W group is much better than the other two groups. Thus, we infer that CAN has different inhibitory effects on fibrosis in rats with different degrees of myocardial fibrosis.

### 3.3. CAN Treatment Decreases the Expressions of AT_1_R and p-FAK of Myocardial Fibrosis Rats

**Figure 3:**
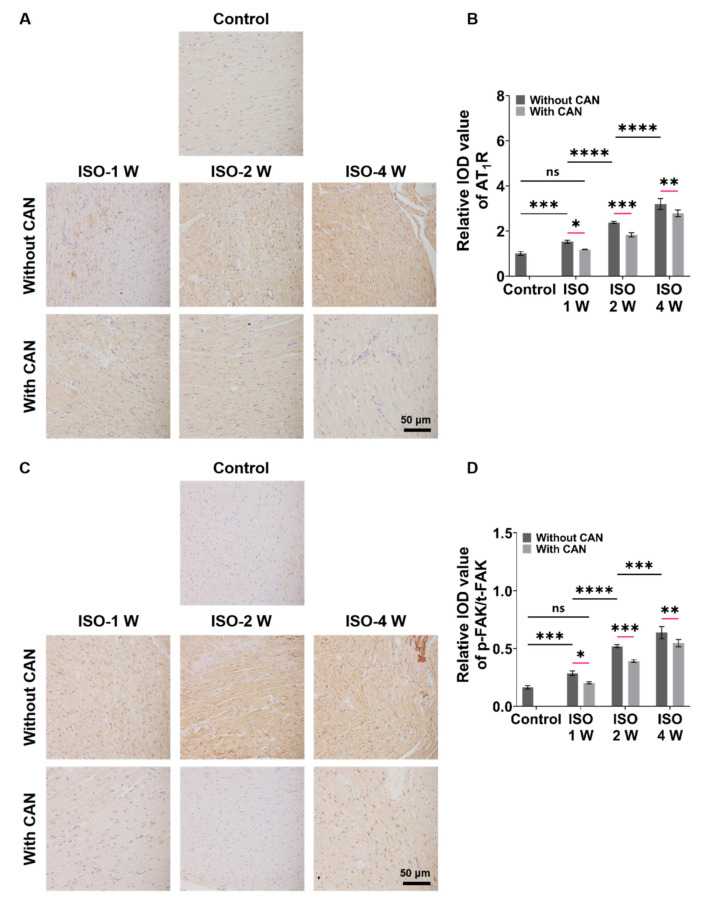
Characterization results of AT_1_R and p-FAK expressions in myocardial fibrosis rats without and with CAN treatment. (**A**) AT_1_R immunohistochemical images of fibrotic myocardium without and with CAN treatment. (**B**) Statistical histograms of relative AT_1_R content of the fibrotic myocardium (*n* = 5). (**C**) p-FAK immunohistochemical images of fibrotic myocardium without and with CAN treatment. (**D**) Statistical histograms of p-FAK/t-FAK of the fibrotic myocardium (*n* = 5). Data are shown as means ± SEM. ns, no significant difference, * *p* < 0.05, ** *p* < 0.01, *** *p* < 0.001, and **** *p* < 0.0001 determined by two-way ANOVA.

The characterizations of the expressions of AT_1_R and p-FAK, two important mechanical signal transduction proteins, are important for understanding the role of mechanical signals in fibrosis. From Figure 3A, we can see that the AT_1_R expressions of the rat heart tissues are up-regulated with the extension of ISO induction time without CAN treatment, while the AT_1_R expressions in the groups with CAN treatment are down-regulated. Moreover, the inhibition efficacy of CAN in the ISO-2 W group is better than those in the ISO-1 W and ISO-4 W groups (Figure 3B). From Figure 3C, we can see that the expression of p-FAK gradually increases with the deterioration of the fibrosis degree in rats, which is similar to AT_1_R without CAN intervention, while the expression of p-FAK decreases after CAN intervention. The t-FAK expressions have no significant difference among the groups of ISO-1W, ISO-2W, and ISO-4W without and with CAN treatment (Supplemental Figure S2). Therefore, we further analyzed the p-FAK/t-FAK ratios of ISO-1 W, ISO-2 W, and ISO-4 W groups without and with CAN treatment, and the results showed that the reduction of the p-FAK/t-FAK ratio of the ISO-2 W group after CAN treatment is greater than those of ISO-1 W and ISO-4 W groups (Figure 3D).

### 3.4. CAN Treatment Ameliorates the Oxidative Stress State of Myocardial Fibrosis Rats

**Figure 4:**
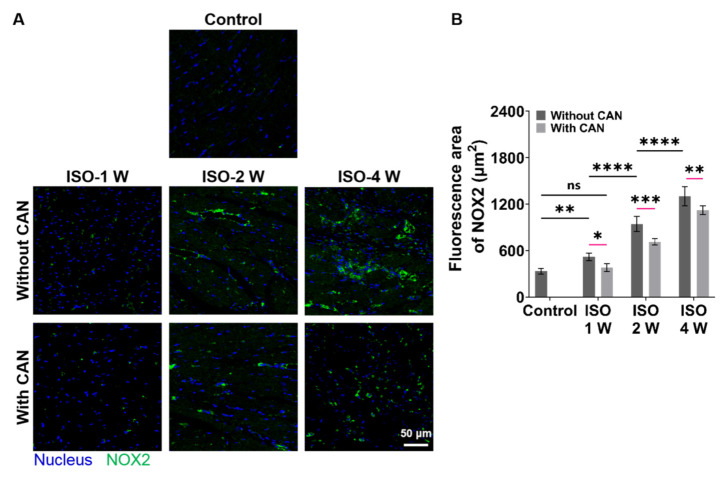
Characterization results of oxidative stress levels in myocardial fibrosis rats without and with CAN treatment. (**A**) NOX2 fluorescence images of fibrotic myocardium without and with CAN treatment. (**B**) Statistical histograms of fluorescence area of NOX2 of the fibrotic myocardium (*n* = 5). Data are shown as means ± SEM. ns, no significant difference, * *p* < 0.05, ** *p* < 0.01, *** *p* < 0.001, and **** *p* < 0.0001 determined by two-way ANOVA.

Since NOX2 is the major source of ROS in the cardiovascular system [30], we checked the oxidative stress levels of rat hearts by measuring the NOX2 expression levels of rat heart tissues. The NOX2 expressions of the rat heart tissues are up-regulated with the extension of ISO induction time, while the NOX2 expressions in the groups with CAN treatment are down-regulated (Figure 4A), which decrease by 21.6%, 26.7%, and 18.2% in the groups of ISO-1 W, ISO-2 W, and ISO-4 W, respectively (Figure 4B). CAN have the best therapeutic efficacy on the oxidative stress state of the rats in the ISO-2 W group among all three groups. The above animal experimental results indicate that the ISO-2 W group with CAN treatment has the best efficacy in myocardial fibrosis by decreasing the myocardial collagen content, plasma markers of myocardial injury, and oxidative stress state, and then improving the cardiac function. We, thus, hypothesize that the different CAN efficacy in myocardial fibrosis can be related to the different ECM stiffness of the damaged myocardial tissues. Next, we further used the in vitro cell models to confirm this.

### 3.5. CAN Treatment Inhibits CF Migration and Proliferation under Different ECM Stiffness

**Figure 5:**
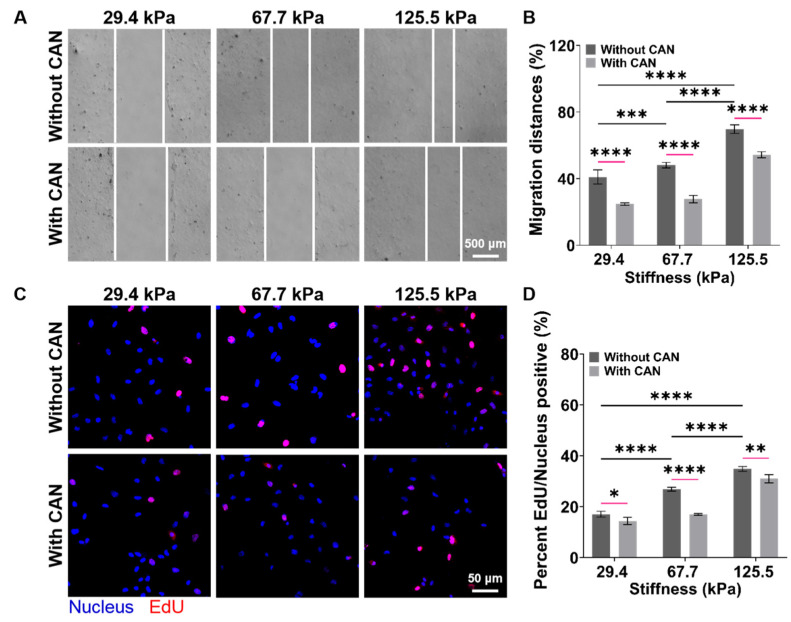
Characterization results of CF migration and proliferation behaviors on the PA gels with a stiffness of 29.4, 67.7, and 125.5 kPa. (**A**) Migration behavior of CFs on PA gels with different stiffness without and with CAN treatment. (**B**) Statistical histogram of CF migration rates (*n* > 3). (**C**) Fluorescence images of CF EdU/Nucleus rates on PA gels with different stiffness without and with CAN treatment. (**D**) Statistical histogram of the CF EdU/Nucleus rates (*n* > 3). Data are shown as means ± SEM. * *p* < 0.05, ** *p* < 0.01, *** *p* < 0.001, and **** *p* < 0.0001 determined by two-way ANOVA.

An in vitro model of the cardiac mechanical microenvironment, which can mimic the structure and mechanical properties of natural heart tissues, has been widely used in cardiovascular disease research and drug screening [31,32]. PA gels have been used for the construction of in vitro cardiac mechanical microenvironment models due to their suitable biological properties and adjustable stiffness [33]. Based on the above in vivo results, we prepared the PA gels with a stiffness of 29.4 ± 4.1, 67.7 ± 5.3, and 125.5 ± 5.7 kPa as the culture substrates of CFs to mimic the early, middle, and late stages of the myocardial fibrosis rats, respectively (Supplemental Figure S3) and used the purified CFs for the subsequent in vitro experiments (Supplemental Figure S4). The CFs migration rates on the PA gels without CAN treatment become faster with increasing PA gel stiffness, and the CFs migration rates with CAN treatment are slower than those without CAN treatment (Figure 5A). The CFs migration rates on the PA gels with a stiffness of 29.4, 67.7, and 125.5 kPa decreased by 16.27%, 20.37%, and 15.43%, respectively (Figure 5B). In addition, from the EdU fluorescence images of the CF on the PA gels (Figure 5C), we observed that the EdU/Nucleus rate increases with increasing PA gel stiffness. The EdU/Nucleus rates on the PA gels decrease with CAN treatment, and the EdU/Nucleus rates on the PA gels with the stiffness of 29.4, 67.7, and 125.5 kPa decrease by 2.18%, 9.97%, and 3.84%, respectively (Figure 5D). These results indicate that the CFs migration and proliferation behaviors without CAN treatment are ECM stiffness-dependent, and CAN can inhibit the CF migration and proliferation induced by ECM stiffness. More importantly, CAN has the most significant inhibitory effect on the CF migration and proliferation rate on the 67.7 kPa PA gels than those on the 29.4 and 125.5 kPa PA gels. Therefore, we consider that the inhibitory effect of CAN on the CF migration and proliferation is affected by the ECM stiffness.

### 3.6. CAN Treatment Inhibits CF Activation and Collagen Protein Production under Different ECM Stiffness

**Figure 6:**
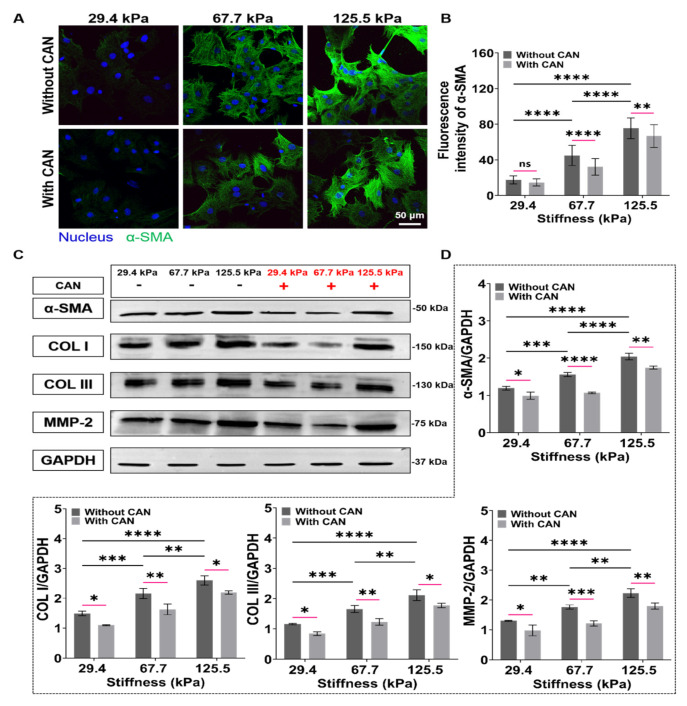
Characterization results of activation and collagen-related proteins of CFs on PA gels with a stiffness of 29.4, 67.7, and 125.5 kPa. (**A**) α-SMA fluorescence images of CFs on PA gels with different stiffness without and with CAN treatment. (**B**) Statistical histogram of α-SMA fluorescence intensities expressed by CFs (*n* > 3). (**C**) Relative protein levels of α-SMA, type I and type III collagens, and MMP-2 of CFs on PA gels with different stiffness without and with CAN treatment determined by western blotting analysis. (**D**) Statistical histogram of protein gray values of α-SMA, type I and type III collagens, and MMP-2 (*n* = 3). Data are shown as means ± SEM. ns, no significant difference, * *p* < 0.05, ** *p* < 0.01, *** *p* < 0.001, and **** *p* < 0.0001 determined by two-way ANOVA.

Upon tissue injury, CFs exposed to high strain or ECM stiffness undergo differentiation into myofibroblasts, in which α-SMA is the most used myofibroblast biomarker and represents the activation degree of CFs [34]. From the α-SMA fluorescence images, we observed that the α-SMA expressions of CFs on the PA gels gradually increase with increasing PA gel stiffness. With CAN treatment, the α-SMA expressions decrease (Figure 6A), and CAN shows better inhibition efficiency on the CF activation on the PA gels with 67.7 kPa than those on the 29.4 and 125.5 kPa PA gels (Figure 6B). The activated myofibroblasts are the main source of collagen in the fibrotic heart, causing a significant increase in type I and type III collagens [35]. In addition, MMP2 is also highly expressed in fibrotic tissues, which also leads to excessive collagen deposition in myocardial tissue [36]. We, thus, used western blotting to further characterize the expressions of α-SMA, type I and type III collagens, and MMP2 of the CFs (Figure 6C), which shows that the type I and type III collagens are up-regulated with the increased PA gel stiffness, accompanied with the increased expressions of α-SMA and MMP2. With CAN treatment, the expressions of α-SMA, MMP-2, type I, and type III collagens are all down-regulated. Additionally, according to the statistical analysis of the grey values of α-SMA, MMP-2, type I, and type III collagen with and without CAN treatment (Figure 6D), the decreased degrees of the grey values on the 67.7 kPa PA gels are more obvious than those on the 29.4 and 125.5 kPa PA gels. Based on the results of α-SMA and collagen production-related proteins, we can find that the increased ECM stiffness intensifies the CF activation and the collagen production, and a significant difference in the inhibition of CF activation and collagen production by CAN. It can thus be speculated that the inhibitory effect of CAN on CF activation and collagen production is also affected by ECM stiffness.

### 3.7. CAN Treatment Ameliorates Redox Imbalance of CFs under Different ECM Stiffness

**Figure 7:**
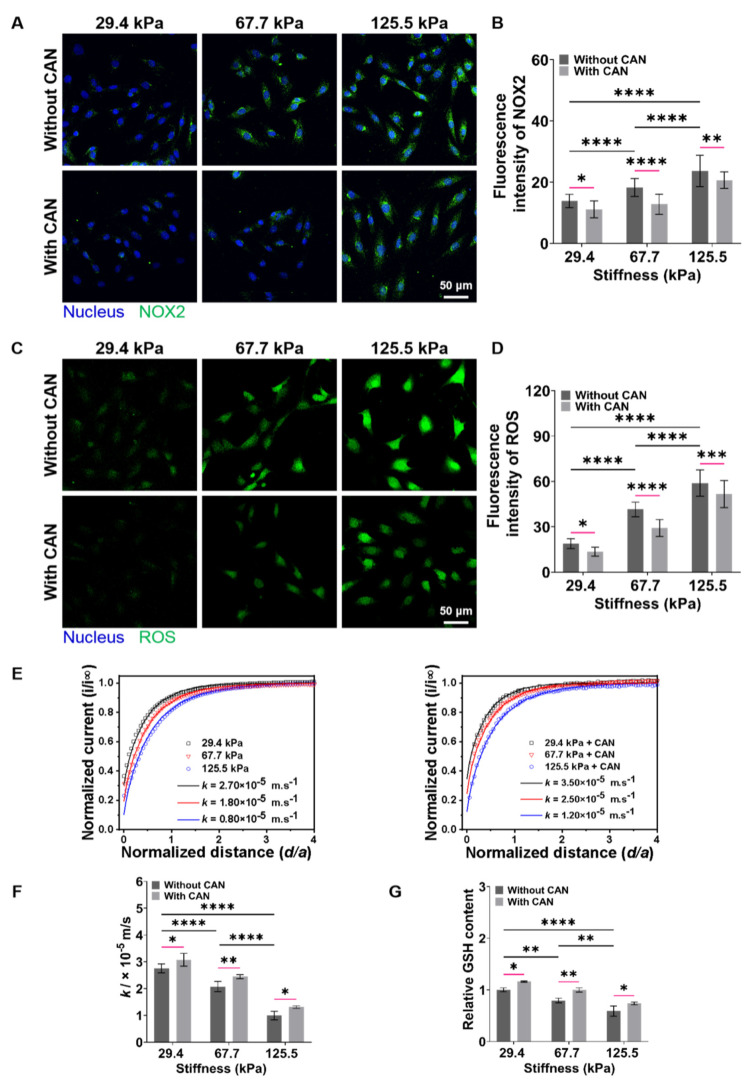
Characterization results of NOX2, ROS, and GSH levels produced from CFs on PA gels with a stiffness of 29.4, 67.7, and 125.5 kPa. (**A**) NOX2 fluorescence images of CFs on PA gels with different stiffness without and with CAN treatment. (**B**) Statistical histogram of NOX2 fluorescence intensities expressed by CFs (*n* > 3). (**C**) ROS fluorescence images of CFs on PA gels with different stiffness without and with CAN treatment. (**D**) Statistical histogram of ROS fluorescence intensities expressed by CFs (*n* > 3). (**E**) SECM approach curves to CFs on PA gels with different stiffness without and with CAN treatment. (*n* = 6). (**F**) Statistical histogram of *k* values of CFs on PA gels with different stiffness without and with CAN treatment (*n* = 6). (**G**) Statistical histogram of relative content of GSH in CFs on PA gels with different stiffness without and with CAN treatment characterized by GSH assay kit (*n* = 3). Data are shown as means ± SEM. * *p* < 0.05, ** *p* < 0.01, *** *p* < 0.001, and **** *p* < 0.0001 determined by two-way ANOVA.

The occurrence and development of myocardial fibrosis are accompanied by the disruption of cellular redox balance [37]. ROS and GSH, the two typical and key cellular redox species play important roles in maintaining cellular redox balance [38]. Herein, we first performed the fluorescence staining experiments to characterize the intracellular NOX2 and ROS levels of the CFs on PA gels. The NOX2 and ROS levels of CFs gradually increase along with the increased PA gel stiffness, while the NOX2 and ROS levels of CFs decrease with CAN treatment (Figure 7A,C). Furthermore, the analysis of the NOX2 and ROS fluorescence intensity shows that the ROS levels produced by the CFs on the 67.7 kPa PA gels with CAN treatment decrease most significantly (Figure 7B,D). It indicates that CAN can effectively reduce the accumulation of oxidative substances of CFs induced by ECM stiffness, and CAN has the most obvious antioxidant function on the CFs on the 67.7 kPa PA gels. Moreover, GSH depletion by its efflux is also taken as a marker of oxidative stress and independently precedes ROS accumulation [39,40], that is, the GSH efflux is also a key biological indicator to evaluate the cell damage and apoptosis. We further characterized the dynamic GSH efflux process across the CFs using SECM.

SECM, an electrochemical scanning probe microscopy with using a micrometer/nanometer-sized electrode as its probe to record the redox currents around living cells in a cell culture medium in an in situ, non-invasive and label-free manner [41,42], has been widely applied to monitor the levels of several chemical substances (e.g., GSH, oxygen, H_2_O_2_) released by living cells [43,44]. In the SECM experiments, we used FcCOOH as the redox mediator and applied the oxidation potential of FcCOOH at the probe to characterize the GSH efflux from CFs on PA gels. In principle, the oxidized [FcCOOH]^+^ at the probe diffuses to the CF surface and reacts with the GSH released by the CF to regenerate FcCOOH, which diffuses back to the probe surface and results in an oxidation current of FcCOOH. When the SECM probe approaches the CF surface, a pure negative feedback current can be obtained, which results from the hindering effect of CF on the FcCOOH diffusion to the probe surface. When approaching the SECM probe to the surfaces of CFs on the stiff PA gels (67.7 and 125.5 kPa), the lower probe oxidation currents of FcCOOH compared with those of the CFs on the soft PA gels (29.4 kPa) are obtained (Figure 7E). It can be due to the less GSH efflux generated from the CFs on the stiff PA gels, thus leading to a smaller recycle oxidation current of FcCOOH compared to those of CFs on the softer PA gels.

Then, based on the recorded average heights and lengths of CFs on the PA gels with different stiffness (Supplemental Figure S5), we built a 2D axial simulation model of the SECM experiments using COMSOL Multiphysics software (Supplemental Figure S6). By fitting the obtained SECM approach curves with the theoretical ones, we can obtain the regeneration rate (*k*) of FcCOOH, which can represent the outflow rate of GSH. From the obtained average *k* values of the CFs on the PA gels through six repeated SECM measurements, we can see a decreasing trend of *k* values with the increased PA gel stiffness, indicating that the less extracellular GSH levels of CFs on the stiff ECM produced. Comparably, the average *k* values of CFs with CAN treatment are higher than those of CFs on the PA gels without CAN treatment. Based on the analysis of the difference in the average *k* values without and with CAN treatment (Figure 7F), we can obtain that the increase of the average *k* values of CFs on the 67.7 kPa PA gels are significantly higher than those on the 29.4 and 125.5 kPa PA gels. These results indicate that CAN can ameliorate the oxidative state of CFs induced by the ECM stiffness, thus reducing GSH consumption, and the GSH consumption state of CFs on the 67.7 kPa PA gels is ameliorated best compared to those on the 29.4 and 125.5 kPa PA gels.

Furthermore, since GSH is the most important biological reductant in cells, it is also important to detect the intracellular GSH content of CFs. The result of the GSH assay kit shows that the intracellular GSH contents decrease with the increased ECM stiffness (Figure 7G). With CAN treatment, the intracellular GSH contents increase, and the intracellular GSH contents in the CFs on the 67.7 kPa PA gels increase most significantly. Additionally, the change in the intracellular GSH contents is consistent with the change in the GSH efflux. Based on the above obtained ROS and GSH level results, we conclude that ECM stiffness can lead to the oxidative stress of CFs, and CAN can alleviate the oxidative stress of CFs induced by ECM stiffness. Moreover, CAN presents the strongest antioxidant efficacy to CFs on the PA gels with a medium stiffness of 67.7 kPa.

### 3.8. ECM Stiffness-Dependent Anti-Fibrosis and Antioxidant Efficacy of CAN by Regulation of AT_1_R and p-FAK

**Figure 8:**
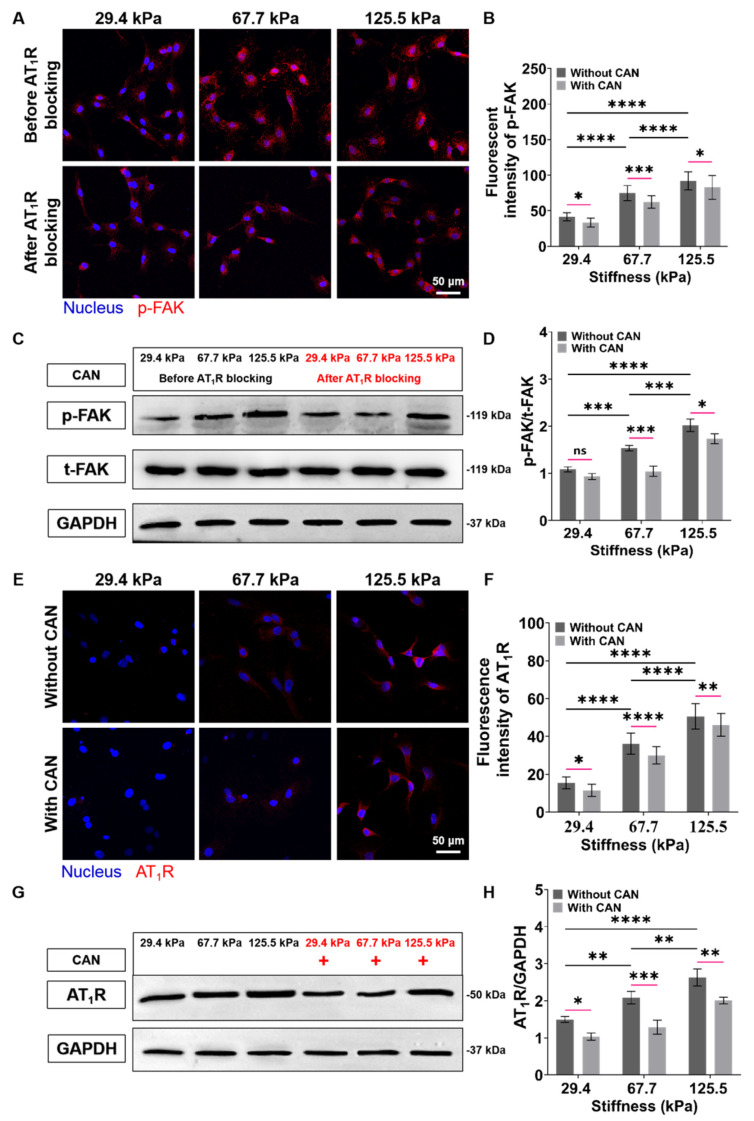
Characterization results of expressions of p-FAK and AT_1_R of CFs on PA gels with a stiffness of 29.4, 67.7, and 125.5 kPa. (**A**) p-FAK fluorescence images of CFs on PA gels with different stiffness before and after AT_1_R blocking. (**B**) Statistical histogram of p-FAK fluorescence intensities expressed by CFs (*n* > 3). (**C**) Relative protein levels of p-FAK of CFs on PA gels with different stiffness before and after AT_1_R blocking determined by western blotting analysis. (**D**) Statistical histogram of protein gray values of p-FAK (*n* = 3). (**E**) AT_1_R fluorescence images of CFs on PA gels with different stiffness without and with CAN treatment. (**F**) Statistical histogram of AT_1_R fluorescence intensities expressed by CFs (*n* > 3). (**G**) Relative protein levels of AT_1_R of CFs on PA gels with different stiffness without and with CAN treatment determined by western blotting analysis. (**H**) Statistical histogram of protein gray values of AT_1_R (*n* = 3). Data are shown as means ± SEM. ns, no significant difference, * *p* < 0.05, ** *p* < 0.01, *** *p* < 0.001, and **** *p* < 0.0001 determined by two-way ANOVA.

To evaluate the role of FAK in ECM stiffness-mediated activation of CFs, we characterized the p-FAK expressions of CFs. We can see from the p-FAK fluorescence images that the p-FAK expressions of CFs increase with the increase of the PA gels stiffness. To determine whether FAK is downstream of AT_1_R in ECM stiffness-induced CF activation, we blocked the signal transduction of CFs mediated by AT_1_R on the PA gels with different stiffness through angiotensin receptor inhibitor and measured the p-FAK expressions of the CFs again. The p-FAK expressions in CFs on the PA gels with a three stiffness with a CAN treatment are reduced (Figure 8A). Subsequently, from the analysis of the decreased levels of the p-FAK expressions without and with the CAN treatment, the p-FAK expressions of the CFs on the 67.7 kPa PA gels decrease most among the three groups (Figure 8B) Similarly, the western blotting results also confirm that ECM stiffness could promote FAK phosphorylation while blocking AT_1_R could reduce the level of p-FAK (Figure 8C,D). These results imply that AT_1_R may function as an important upstream molecule to mediate FAK-dependent CF mechanotransduction. Therefore, we speculate that the best CAN efficacy on CFs on the 67.7 kPa PA gels is due to the most obvious decrease of p-FAK in CFs on the 67.7 kPa PA gels, which may be related to the expression of AT_1_R. We further characterized the expression of AT_1_R under the combined effects of ECM stiffness and CAN treatment. The results of fluorescence images confirm that the mechanical regulation of ECM stiffness directly leads to the increase of AT_1_R expression. While CAN inhibits the AT_1_R expression, which is consistent with the pharmacological effect of CAN (Figure 8E). From the analysis of the changes in AT_1_R expression in CFs on the PA gels with different stiffness with CAN treatment in Figs. 8F, the decrease of the AT_1_R activities in CFs on the 67.7 kPa PA gels with CAN treatment is the most obvious. The western blotting results in Figure 8G,H also confirm the above changes in AT_1_R.

**Figure 9:**
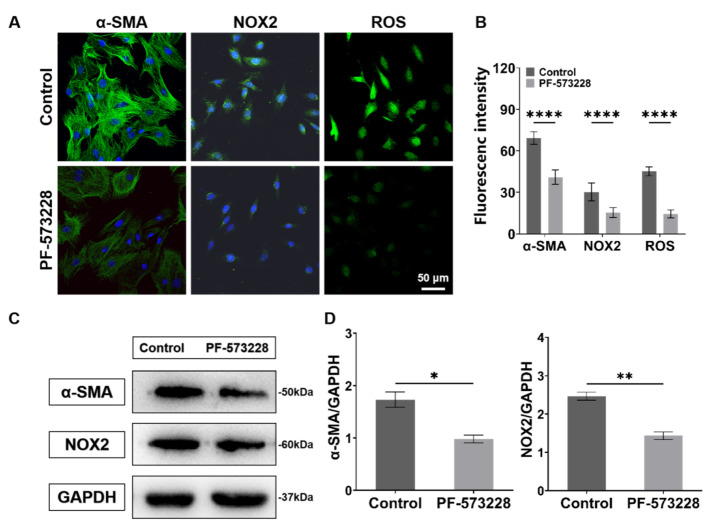
Characterization results of expressions of α-SMA, NOX2, and ROS of CFs on PA gels with PF-573228 intervention. (**A**) α-SMA, NOX2, and ROS fluorescence images of CFs with PF-573228 intervention. (**B**) Statistical histogram of fluorescence intensities of α-SMA, NOX2, and ROS (*n* > 3). (**C**) Relative protein levels of α-SMA and NOX2 of CFs with PF-573228 intervention determined by western blotting analysis. (**D**) Statistical histogram of protein gray values of α-SMA and NOX2 (*n* = 3). Data are shown as means ± SEM. * *p* < 0.05, ** *p* < 0.01, and **** *p* < 0.0001 determined by paired *t*-test.

Next, to study the role of FAK in ECM-mediated fibroblast activation and cellular oxidative stress. We treated CFs with the FAK inhibitor (PF-573228, 10 μM; MCE, HY-10461). The obtained fluorescence and western blotting results both show that the expressions of α-SMA, NOX2, and ROS significantly decreased after the PF-573228 intervention (Figure 9), indicating that FAK can regulate the activation and redox state of CFs.

**Figure 10:**
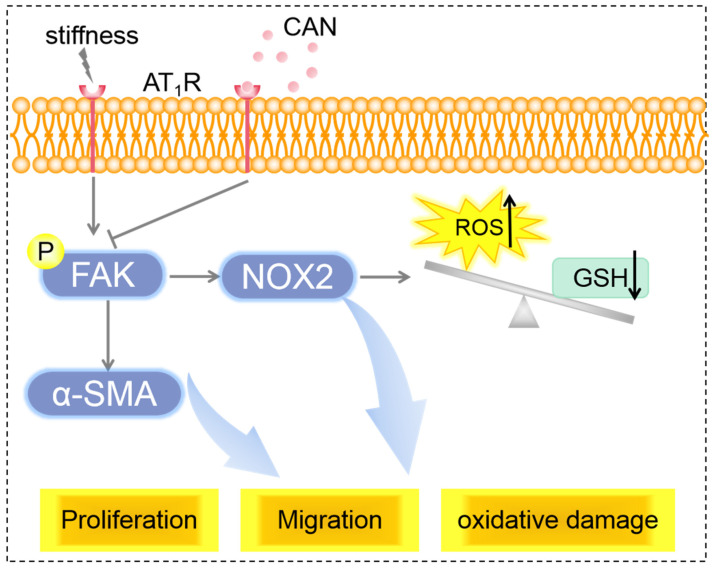
Scheme of mechanisms of ECM stiffness effect on CAN efficacy in myocardial fibrosis.

From the above results, we can conclude that the co-regulation of ECM stiffness and CAN efficacy lead to the difference in the AT_1_R expression of CFs on the PA gels with three stiffness, which is manifested by the most obvious decline of AT_1_R expression of CFs on the 67.7 kPA gels. The difference in the AT_1_R expression regulates the difference in the reduction of p-FAK, further leading to the different improvements of CF activation and oxidative stress state (Figure 10).

## 4. Discussion

ECM is a linchpin of myocardial tissue with the functions of maintaining structural and functional integrity and providing the ambient microenvironment required for mechanical, cellular, and molecular activities in the heart. Myocardial fibrosis would inevitably result in profound changes in the composition and structure of ECM, such as collagen deposition, increased stiffness, and impaired contraction. The stiffness of native myocardial tissues at the adult stage is only 10–20 kPa, while the stiffness of myocardial tissue can increase from 30 to 90 kPa for post-myocardial infarction. Considering that the ventricular remodeling induced by ISO in rats is a classic model for studying myocardial fibrosis, we chose the ISO-induced rat myocardial fibrosis model in our case and we found that the myocardial tissue stiffness of myocardial fibrosis (125.5 kPa) is higher accompanied by the progressive deterioration of cardiac function, from which we can see a positively correlated relationship between the stiffness of myocardial tissue and the degree of fibrosis within the physiological and pathological ranges of fibrosis. This proves that the increase of ECM stiffness and the injury of cardiac function promote each other and the myocardium stiffness can be a valuable new metric for determining the cardiac dysfunction in patients with heart disease and reflecting the development and prognosis of myocardial fibrosis to a certain extent [45].

The concept of “biomechanopharmacology” has been introduced in 2002, which is a new discipline established by combining biomechanics/mechanobiology and pharmacology [46]. It not only focuses on the influence of biomechanical factors on pharmacological effects but also emphasizes the change in drug efficacy by changing biomechanical events [47,48]. For instance, phosphoinositide 3-kinases (PI3Ks) β isoform exerts a significant role under high shear stress by transferring the force of actin cytoskeleton in activated platelets to clotting fibrin through platelet integrin, thus inhibiting the action of anticoagulants [49]. Matrix stretching can activate transient receptor potential cation channel V4 (TRPV4), and preclinical animal studies have successfully shown that oral TRPV4 channel inhibitors can prevent pulmonary edema relevant to heart failure [50]. In our study, the myocardial tissues of rats with medium stiffness and the CFs on the 67.7 kPa PA gels present the most obvious declining trend of fibrosis after CAN treatment. The high ECM stiffness weakens the efficacy of CAN for anti-fibrosis, which may be due to that the mechanical signal promotes the progress of fibrosis through mechanical pathways (e.g., integrin). For the rats in the ISO-1W group, the degree of myocardial fibrosis is relatively mild, and the CAN efficacy relatively declines which might be due to the self-healing mechanisms of heart. The CFs on the 29.4 kPa PA gel show no significant increase in the stiffness-induced AT_1_R expression and a relative decline in the CAN efficacy for CFs on the 29.4 kPa PA gels. FAK, an enzyme widely existing in the cytoplasm, plays a vital role in various types of fibrosis [18]. Phosphatidylinositol 3-kinase/protein kinase B (PI3K/Akt) and ERK1/2, the downstream of FAK, are the two classical signaling factors that directly cause myocardial fibrosis [51,52], and can result in the α-SMA-positive myofibroblast diversity and the formation of various types of collagens. FAK also directly regulates the expression of α-SMA, and thus participates in the process of liver fibrosis [53]. the expression of AT_1_R can be induced by ECM stiffness, and the expression level of AT_1_R increases with the increase of ECM stiffness, demonstrating the activation effect of ECM stiffness on AT_1_R. For the group with CAN treatment, CAN leads to the decrease of AT1R expression, which shows the opposite effect to the increase of AT_1_R expression induced by ECM stiffness. It can be due to that CAN can selectively and non-competitively bind AT_1_R, resulting in the decreased expression of AT_1_R. In addition, our results also prove the upstream and downstream relationship between p-FAK and AT_1_R, i.e., AT_1_R is an upstream protein of FAK and mediates ECM stiffness-induced CF activation through FAK. Therefore, the resistance effect between the pharmacological efficacy of CAN and the ECM stiffness influence leads to the difference in AT_1_R expression of CFs on the PA gels with different stiffness, further leading to the change of downstream p-FAK expression, which is also the reason for the difference in the CAN efficacy. Our results also prove that AT_1_R can only regulate FAK to a certain extent, which can be due to that FAK as a downstream molecule is also affected by other membrane mechanosensitive proteins (e.g., integrins). However, the differences in drug efficacy caused by the complex mechanical regulation mechanisms and biochemical factors deserve further analysis and discussion.

Oxidative stress injury is one of the main features of fibrosis. The elevation of NOX2 and ROS [54] and the reduction of GSH synthesis have been proven in organ fibrosis [55] and cardiovascular disease [56]. Moreover, the GSH efflux regulates the redox state in the extracellular microenvironment and cooperates with the intracellular redox status regulation system to maintain cellular redox homeostasis [57]. Our results confirm that ECM stiffness can directly lead to the accumulation of NOX2, and ROS and the reduction of intracellular and extracellular GSH. It is also noted that CAN has the most obvious antioxidant efficacy under the mechanical condition of medium stiffness. Some studies showed that ECM stiffness can directly activate NOX2 to produce ROS by affecting the cellular microtubule function [58]. Our experimental results also prove that ECM stiffness itself can regulate the expressions of NOX2 and ROS, which is regulated by FAK. Therefore, we consider that ROS changes may also be attributed to the FAK regulation by the ECM stiffness. In addition, the characterization results of GSH in our work show that both the intracellular GSH content and the GSH efflux decrease with the increased ECM stiffness, which is opposite to the change in the ROS level of CFs. Since the consumption of GSH is mainly related to the accumulation of oxidative stress substances inside and outside cells, we can thus speculate that the change trend of the intracellular GSH content and the GSH efflux of CFs may be related to the accumulation of ROS. However, the direct regulation of GSH by ECM stiffness needs further research.

## 5. Conclusions

The main finding of this study is that ECM stiffness has a certain impact on the CAN efficacy in anti-fibrosis and antioxidative efficacy, and CAN shows the obvious antioxidative and anti-fibrosis efficacy at the medium-stiffness (67.7 kPa) range of ECM. First, we constructed the rat models with three different myocardial fibrosis degrees and confirmed the difference in the anti-fibrosis and antioxidant efficacy of CAN in the hearts with different stiffness. Then, using the in vitro ECM stiffness-mediated myocardial fibrosis models constructed on the stiffness-adjustable PA gels, the differences in the therapeutic efficacy of CAN on the functions of CFs under different ECM stiffness were identified. Finally, based on the p-FAK and AT1R expression results, we considered that the difference in CAN efficacy is related to the difference in AT_1_R-FAK activity co-regulated by ECM stiffness and CAN. This study can contribute to a better understanding of the interaction between ECM stiffness and the efficacy of CAN in treating myocardial fibrosis, deepening the understanding of the physiological role of ECM stiffness in vivo and calls for attention to the weakening effect of mechanical factors on drug efficacy in the clinical treatment of myocardial fibrosis. We can, thus, speculate that blocking mechanical signals can be a strategy for the treatment of myocardial fibrosis.

## Figures and Tables

**Table 1:** Grouping and treatment in rats.

Group	ISO (Day)	CAN (Day)
1. ISO-1 W	From day 1–day 7	/
2. ISO-2 W	From day 1–day 14	/
3. ISO-4 W	From day 1–day 28	/
4. ISO-1 W+CAN	From day 1–day 7	From day 1–day 28
5. ISO-2 W+CAN	From day 1–day 14	From day 1–day 28
6. ISO-4 W+CAN	From day 1–day 28	From day 1–day 28

## Data Availability

The data is contained within the article or Supplementary Materials.

## Supplementary Materials

The following supporting information can be downloaded at: https://www.mdpi.com/article/10.3390/antiox12030679/s1, Supplementary figures: Figure S1: Cardiac anatomy photos and heart weight indexes of myocardial fibrosis rats without and with CAN treatment; Figure S2: The t-FAK immunohistochemical image of fibrotic myocardium. Figure S3: Young’s modulus of elasticity of the PA gels; Figure S4: Optical microscope image of CFs and fluorescent images of vimentin protein expressions in CFs. S2. Topography of cardiac fibroblasts in SECM measurements; Figure S5: Distributions of heights and lengths of CFs cultured on PA gels with stiffness of 29.4, 67.7 and 125.5 kPa without and with CAN treatment. S3. SECM simulation model; Figure S6: Schematic diagram of SECM simulation model. S4. Experimental methods.
