# Supplementary material for: Effect of Extracellular Matrix Stiffness on Candesartan Efficacy in Anti-Fibrosis and Antioxidation
Source: Antioxidants (Basel). 2023 Mar 9;12(3):679. doi: 10.3390/antiox12030679 (PMC10044920; doi:10.3390/antiox12030679)
Supplement: Supplementary file 1 [file antioxidants-12-00679-s001.zip › Supplementary Materials.pdf]

Supplementary Materials

Effect of Extracellular Matrix Stiffness on Candesartan Efficacy in Anti-Fibrosis and Antioxidation

Tong Zhu [1,2,3], Jingjing Song [1,2], Bin Gao [4], Junjie Zhang [1,2], Yabei Li [2,5], Zhaoyang Ye [1,2], Yuxiang Zhao [1,2], Xiaogang Guo [6], Feng Xu [1,2] and Fei Li [1,2,*]

[1] The Key Laboratory of Biomedical Information Engineering of Ministry of Education, School of Life Science and Technology, Xi'an Jiaotong University, Xi'an 710049, China; tongzhu@stu.xjtu.edu.cn (T.Z.); jingjingsong@stu.xjtu.edu.cn (J.S.); junjie-zhang@stu.xjtu.edu.cn (J.Z.); yzy3119113038@stu.xjtu.edu.cn (Z.Y.); yuxiangzhao@stu.xjtu.edu.cn (Y.Z.); fengxu@mail.xjtu.edu.cn (F.X.)

[2] Bioinspired Engineering and Biomechanics Center (BEBC), Xi'an Jiaotong University, Xi'an 710049, China; liyabei@stu.xjtu.edu.cn

[3] Department of Cardiovasology, Xidian Group Hospital, Xi'an 710077, China

[4] Department of Endocrinology, Tangdu Hospital, Air Force Military Medical University, Xi'an 710032, China; bingao@fmmu.edu.cn

[5] School of Chemistry, Xi'an Jiaotong University, Xi'an 710049, China

[6] Department of Cardiology, The First Affiliated Hospital, Zhejiang University School of Medicine, Hangzhou 310003, China; gxg22222@zju.edu.cn

* Correspondence: feili@mail.xjtu.edu.cn

Table of Contents

S1. Supplementary figures **S3**

S1.1. Figure S1 .... S3

S1.2. Figure S2 .... S3

S1.3. Figure S3 .... S4

S1.4. Figure S4 .... S4

S2. Topography of cardiac fibroblasts in SECM measurements **S4**

S3. SECM simulation model **S6**

S4. Experimental methods **S7**

S4.1. Assessment of cardiac functions of rats S7

S4.2 Assessment of body weight and heart weight of rats S8

S4.3. Staining Masson's Trichrom S8

S4.4. Purity identification of cardiac fibroblasts S8

S4.5. Scratching assay for neonatal rat cardiac fibroblast (NRCF) migration...... S9

S4.6. EdU incorporation assay S9

S4.7. Detection of intracellular ROS levels S9

S4.8. Detection of intracellular GSH levels S9

References **S10**

S1. Supplementary figures

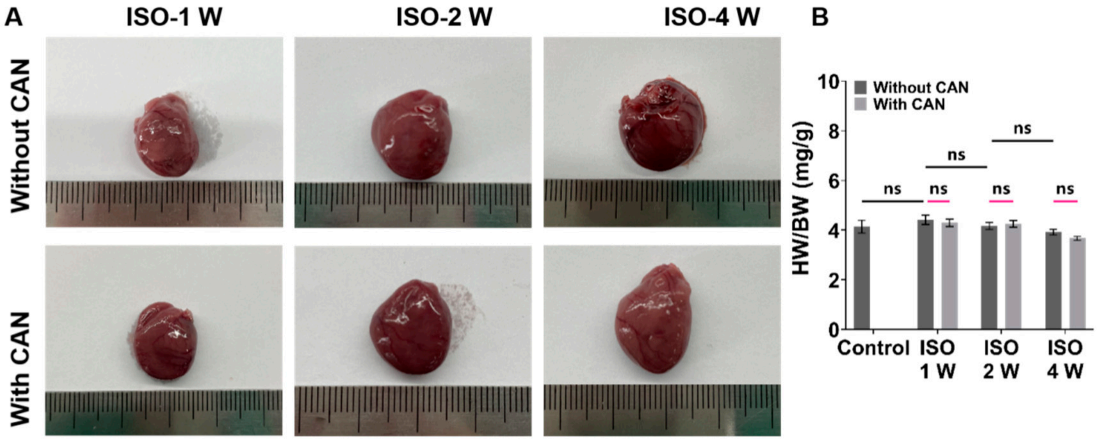

Figure S1. (A) Cardiac anatomy photos and (B) heart weight indexes of myocardial fibrosis rats without and with CAN treatment (*n* = 5). Data are shown as means ± SEM.

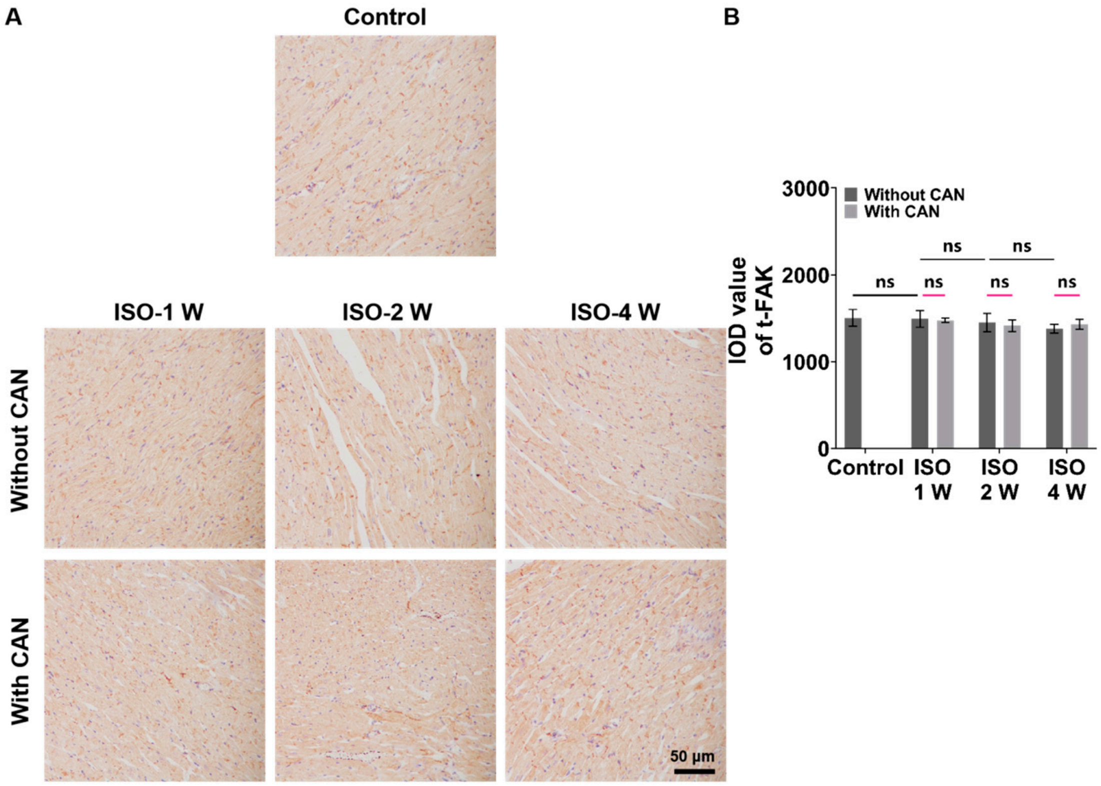

Figure S2. (A) t-FAK immunohistochemical image of fibrotic myocardium. (B) Statistical histograms of relative t-FAK content of the fibrotic myocardium (*n* = 5). Data are shown as means ± SEM.

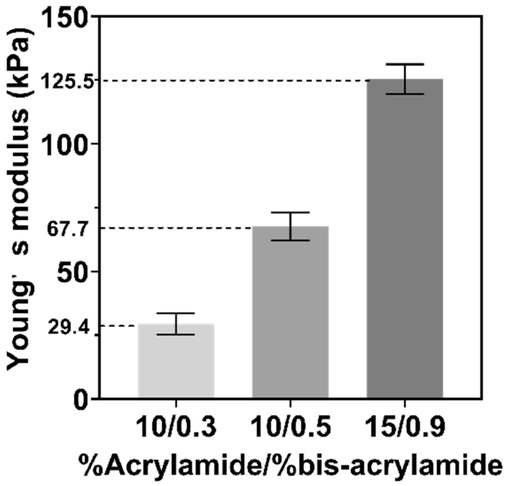

Figure S3. Young's modulus of elasticity of the PA gels *(n*>3).

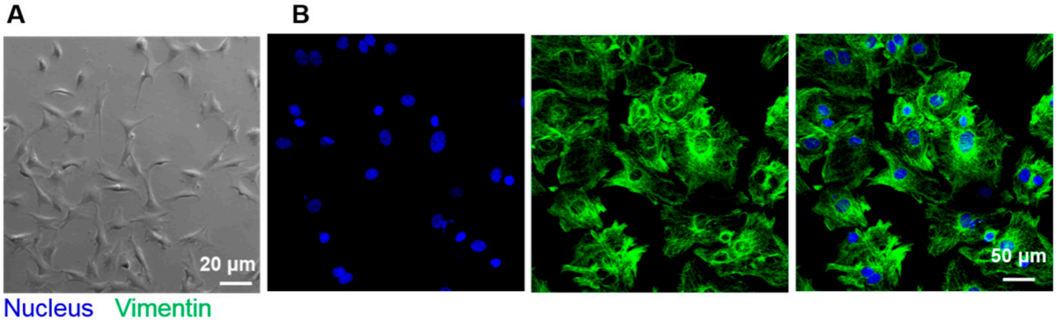

Figure S4. (A) Optical microscope image of cardiac fibroblasts. (B) Fluorescent images of vimentin protein expressions in cardiac fibroblasts (*n*>3).

S2. Topography of cardiac fibroblasts in SECM measurements

Considering that the heights and spreading areas of the cardiac fibroblasts (CFs) on the PA gels with different ECM stiffness are the key parameters in our SECM simulation model[1], we used SECM to characterize the heights and lengths (long axis of cell) of the CFs on the PA gels with three stiffness. As shown in **Figure S5**, the heights and lengths of the CFs on the 29.4, 67.7 and 125.5 kPa PA gels without CAN treatment are 4.55±1.44 μm and 50.45±5.72 μm, 3.65±1.50 μm and 58.63±10.25 μm, 2.69±1.45 μm and 64.31±13.70 μm, respectively. The heights and lengths of the CFs on the 29.4, 67.7 and 125.5 kPa PA gels with CAN treatment are 4.35±1.30 μm and 50.03±6.84 μm, 3.65±1.31 μm and 54.63±10.14 μm, 2.63±1.04 μm and 59.86±13.96 μm, respectively. These results show that all the heights and lengths of the CFs on the PA gels with three stiffness are different and follow normal distributions. The differences in the heights and lengths of the CFs are not obvious

between the groups with CAN treatment and without CAN treatment. Based on these results, we then built the SECM simulation models with including the average heights and lengths of the CFs on the PA gels with different stiffness for simulation with the SECM experimental approach curves.

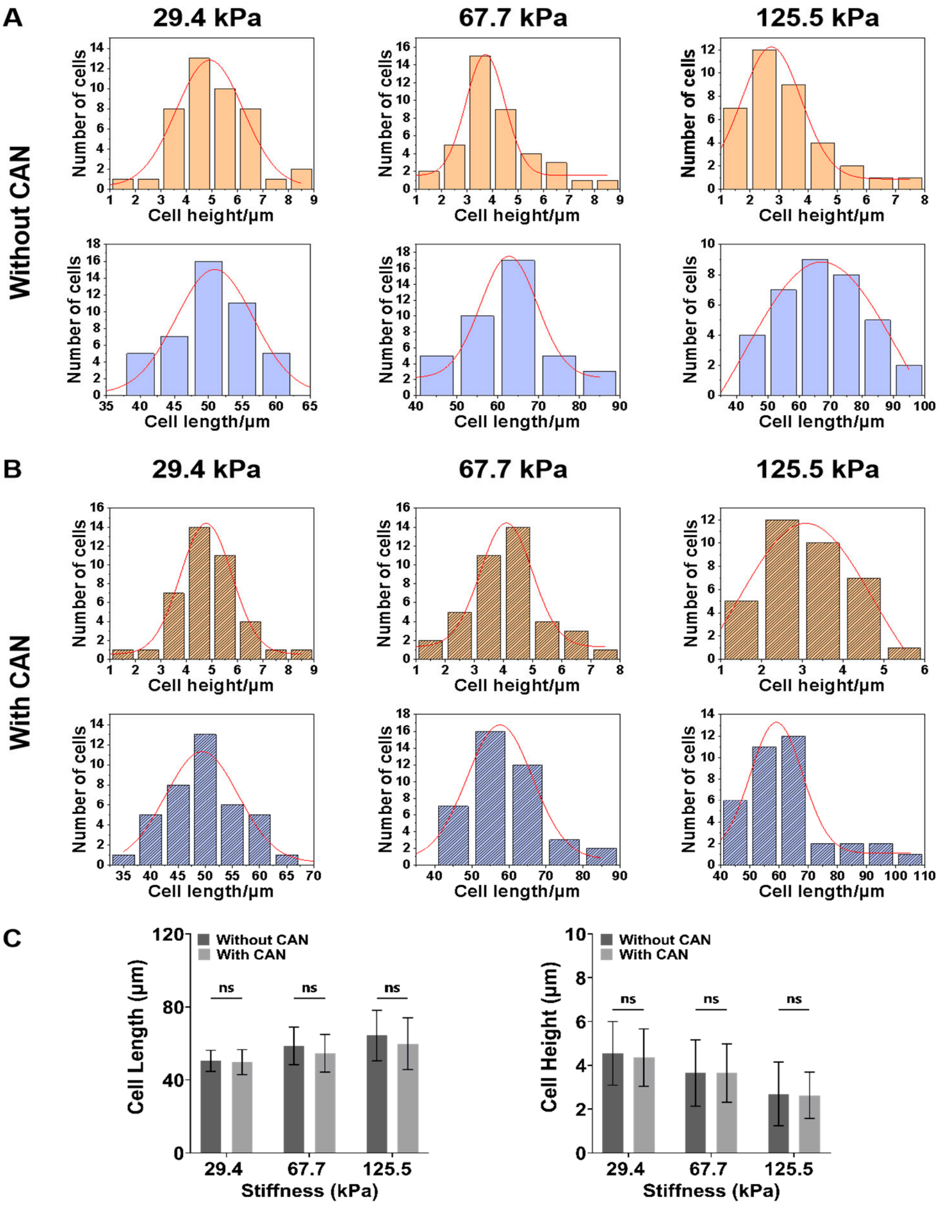

Figure S5. Distributions of heights and lengths of CFs cultured on PA gels with stiffness of 29.4, 67.7 and 125.5 kPa (A) without and (B) with CAN treatment (n = 30). (C) Statistical histograms of heights and lengths of CFs on PA gels with three stiffness without and with CAN treatment (n = 30). ns, no significant difference determined by two-way ANOVA.

S3. SECM simulation model

According to the method previously reported from our group[2], a 2D axial simulation model of SECM was developed with using COMSOL Multiphysics software (COMSOL Inc., Sweden). As illustrated in **Figure S6**, *r* and *z* axes are parallel and perpendicular to the surface of Pt disk electrode, respectively. The origin of the coordinate axes is set at the middle of the Pt disk electrode.

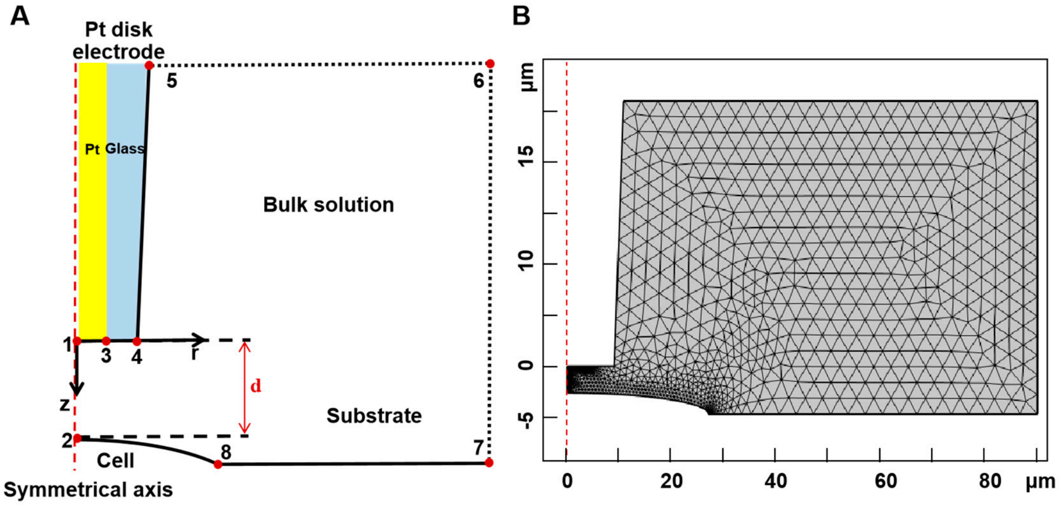

Figure S6. (A) Schematic diagram of SECM simulation model in 2D axial symmetry. (B) A typical meshed 2D SECM model.

In the bulk solution, FcCOOH is oxidized to $[FcCOOH]^+$ at the Pt microelectrode surface (Eq. (S1)). The probe-produced $[FcCOOH]^+$ diffuses to the CF surface and is reduced to FcCOOH by the cell-released GSH (Eq. (S2)).

$$\mathrm{FcCOOH} \rightarrow [\mathrm{FcCOOH}]^+ + e^- \text{ (at the Pt microelectrode surface)} \tag{S1}$$

$$[\mathrm{FcCOOH}]^+ + \mathrm{GSH} \xrightarrow{k} \mathrm{FcCOOH} + \mathrm{GSSG} \text{ (at the cell surface)} \tag{S2}$$

In the model, the diffusions of $[FcCOOH]^+$ and FcCOOH follow Fick's law of diffusion (Eq. (S3)).

$$\frac{\partial c(r,z)}{\partial t} = D\left[\frac{\partial^2 c(r,z)}{\partial r^2} + \frac{1}{r}\frac{\partial c(r,z)}{\partial r} + \frac{\partial^2 c(r,z)}{\partial z^2}\right] = 0 \tag{S3}$$

where *r* and *z* are the coordinate axes, *t* is the time, and $c(r, z)$ is the local concentration of FcCOOH, whose initial condition is given by

$$c(r,z) = c_0, \ t = 0 \tag{S4}$$

Under the diffusion controlled process, the probe redox current can be calculated by Eq. (S5).

$$i_T = 4nFDc_0a = 2\pi FD \int_0^a r \left[\frac{\partial c(r,0)}{\partial z}\right] dr \quad \text{(S5)}$$

where F is the Faraday constant, D is the diffusion coefficient of FcCOOH in aqueous solution (D = 5.7 × 10^{-6} cm^2 s^{-1} in this case), n is the electron transfer number (n = 1 in this case), and a is the radius of the SECM probe (a = 5 μm in this case). The reduction of $[FcCOOH]^+$ by GSH on the cell surface is considered irreversible. The boundary condition at the cell surface can be expressed by

$$D\frac{\partial c(r,z)}{\partial z} = k[c_0 - c(r,d)] \quad \text{(S6)}$$

where d is the distance between the probe and the highest point of the cell. The corresponding coordinates of the points in **Figure S5** are shown in **Table S1**.

Table S1. Definition of the boundary conditions in the 2D SECM simulation model

Boundary		Definition	Equation
**z axes**	**(1→2)**	$\frac{\partial c(r,z)}{\partial r} = 0$, $0<z<d$, $r = 0$	(S7)
**Pt probe surface**	**(1→3)**	$c(r,z) = 0$, $0<r<r_{tip}$	(S8)
**Glass of probe**	**(3→4)**	$\frac{\partial c(r,z)}{\partial z} = 0$, $r_{tip}<r<r_s$, $z = 0$	(S9)
**Glass of probe**	**(4→5)**	$\frac{\partial c(r,z)}{\partial z} = 0$, $\frac{\partial c(r,z)}{\partial r} = 0$, $r_s<r<r_b$	(S10)
**Bulk solution**	**(5→6)**	$c(r,z) = c_0$, $r_b<r<r_m$, $z = h_1$	(S11)
**Bulk solution**	**(6→7)**	$c(r,z) = c_0$, $h_1<z< d + h_{cell}$, $r = r_m$	(S12)
**Cell surface**	**(2→8)**	$D\frac{\partial c(r,z)}{\partial z} = k_f\,[c_0 - c(r,-d)]$, $0<r<r_{cell}$, $z = d$	(S13)
**Substrate**	**(7→8)**	$\frac{\partial c(r,z)}{\partial z} = 0$, $r_{cell}<r<r_m$, $z = d + h_{cell}$	(S14)

S4. Experimental methods

S4.1. Assessment of cardiac functions of rats

All the rats were anesthetized by inhalation of 2% isoflurane (0.8–1.2 L/min). Cardiac structure and function, including the left ventricular internal diameter at end-diastole (LVIDd, mm), left ventricular posterior wall thickness at end-diastole (LVPWd, mm),

interventricular septum thickness at end-diastole (IVSTd, mm), ejection fraction (EF, %), and fractional shortening (FS, %) were assessed using an ultrasonic diagnostics instrument (IE33, Philips, Netherlands) equipped with an S12-4 linear array ultrasound transducer.

S4.2. Assessment of body weight and heart weight of rats

All rats were sacrificed by subcutaneous injection of 2% pentobarbital sodium (200 mg/kg). Then their hearts were removed and weighed. The heart weight index (HWI, in mg/g) was calculated by dividing the heart weight by the body weight.

S4.3. Staining Masson's Trichrome

The myocardial tissues were fixed in 4% paraformaldehyde for 48 h, dehydrated and embedded in paraffin, sliced into 5-μm-thick sections and mounted on glass slides. The slices were stained with a Masson's trichrome staining kit (Servicebio, Wuhan, China), through which the collagen fibers were stained in blue and the myocardium was stained in red. The Quant Center2.1 system (3DHISTECH, Hungary) was used to read the tissue measurement area and calculate the proportion of positive area. Five heart samples were collected from each group of fibrosis rats, and three sections were randomly selected for each heart to calculate the ratios of collagen fiber positive areas. And the ratios of the stained fibrotic areas to the total ventricular areas were calculated and used as the collagen volume fraction.

S4.4. Purity identification of cardiac fibroblasts

Neonatal rat cardiac fibroblasts (NRCFs) were isolated with the differential adhesion method. The cell suspension was inoculated in a culture bottle and placed it in a 37°C, 5% CO_2 incubator for 20 min. Then, the cells adhered to the bottom of the culture bottle were observed using an inverted optical microscopy. The NRCFs were obtained through culturing in 5 mL DMEM/F12 (Corning, Manassas, USA) medium for 20 min and removing the supernatant for three times. Immunofluorescence staining of vimentin protein expression in cardiac fibroblasts (CFs) was used to assess the purity of CFs after NRCFs culturing on a Petri dish for three days.

S4.5. Scratching assay for neonatal rat cardiac fibroblast (NRCF) migration

Glass strips (length × width: 20 mm × 1 mm) were pressed on the PA gel surface, and 1×10^5 NRCFs were inoculated onto the PA gels with different stiffness. After incubating NRCFs until confluency was achieved, the glass strips were removed. The cell monolayer was washed with a serum-free medium to remove the detached cells. Then, the NRCFs were cultured in a complete medium supplemented with or without CAN (1×10^{-6} mol L^{-1}). The NRCFs were photographed at 0 h and 24 h by using a microscope (Eclipse TE2000-U, Nikon, Japan). The widths of the scratches were measured using ImageJ software.

S4.6. EdU incorporation assay

NRCFs were cultured on the PA gels with different stiffness and stimulated with or without the addition of 10^{-6} mol L^{-1} CAN for 5 days. Cell proliferation was determined using an EdU kit (5-ethynyl-2'-deoxyuridine in PBS, Solarbio, Beijing, China) following the manufacturer's instructions. The stained cells were examined and photographed with a laser scanning confocal microscope (FV3000 Olympus, Tokyo, Japan). The proliferation rate of cells was assessed as the proportion of EdU-positive nuclei (red) to blue fluorescence nuclei.

S4.7. Detection of intracellular ROS levels

The ROS/superoxide detection assay kit (1:2000 dilution; Abcam, ab139476) was used to stain the cellular ROS. The cells were incubated with a fluorescent dye at 37°C for 30 min. Images of the cell samples were obtained using a laser scanning confocal microscope (FV3000 Olympus, Tokyo, Japan).

S4.8. Detection of intracellular GSH levels

The Micro Reduced Glutathione (GSH) Assay Kit (Solarbio, Beijing, China) was used to measure the intracellular GSH levels of NRCFs according to the manufacturer's instructions. The absorbance of the sample at 412 nm was recorded using a Spark 10 M Multimode Microplate Reader (TECAN, Switzerland).

References

1. Li, Y.; Lang, J.; Ye, Z.; Wang, M.; Yang, Y.; Guo, X.; Zhuang, J.; Zhang, J.; Xu, F.; Li, F. Effect of Substrate Stiffness on Redox State of Single Cardiomyocyte: A Scanning Electrochemical Microscopy Study. Anal. Chem. 2020, 92, 4771–4779. https://doi.org/10.1021/acs.analchem.9b03178.

2. Lang, J.; Li, Y.; Ye, Z.; Yang, Y.; Xu, F.; Huang, G.; Zhang, J.; Li, F. Investigating the Effect of Substrate Stiffness on the Redox State of Cardiac Fibroblasts Using Scanning Electrochemical Microscopy. Anal. Chem. 2021, 93, 5797–5804. https://doi.org/10.1021/acs.analchem.0c05284.
