# Supplementary figures and images for: Effect of Extracellular Matrix Stiffness on Candesartan Efficacy in Anti-Fibrosis and Antioxidation
Source: Antioxidants (Basel). 2023 Mar 9;12(3):679. doi: 10.3390/antiox12030679 (PMC10044920; doi:10.3390/antiox12030679)

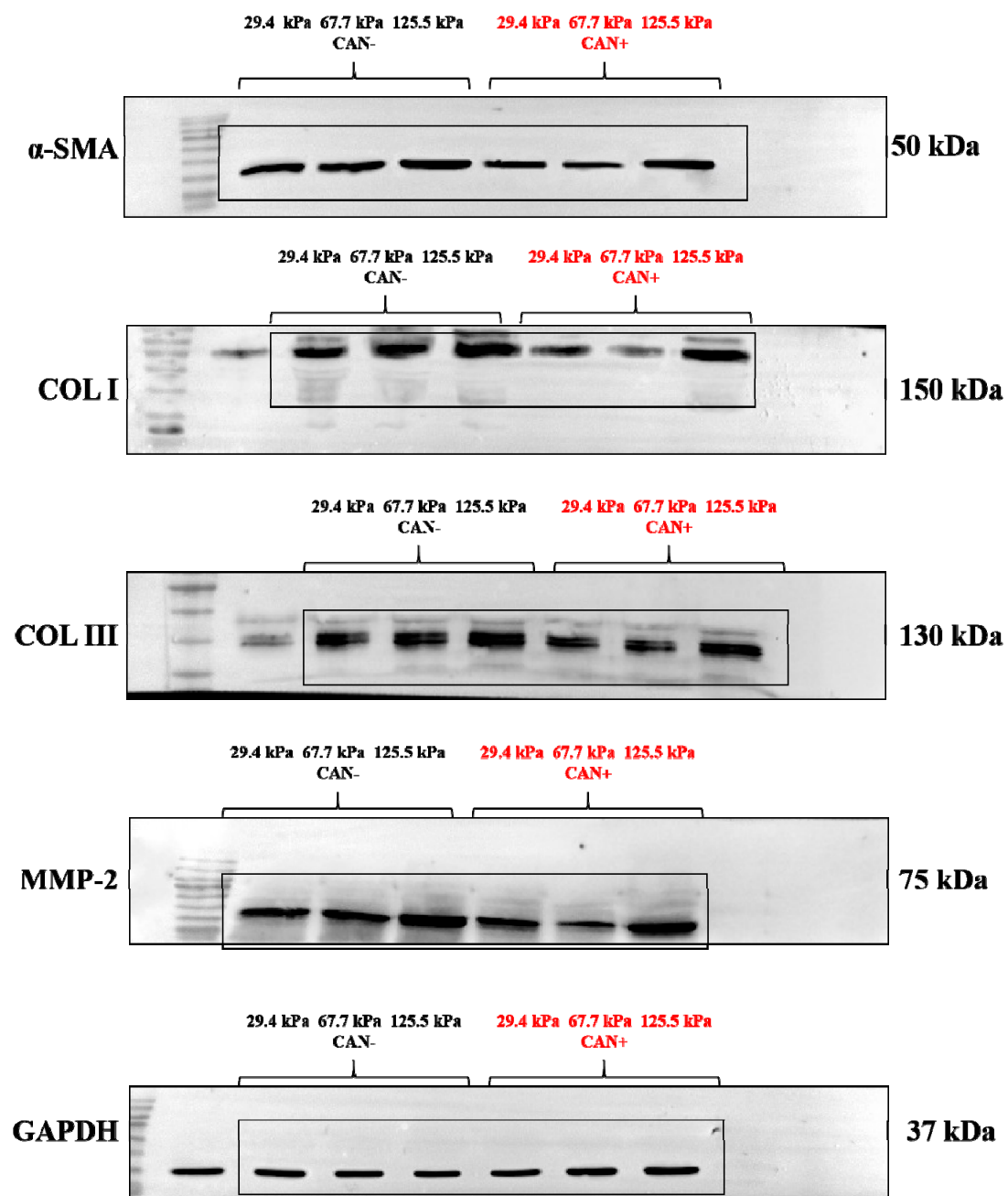

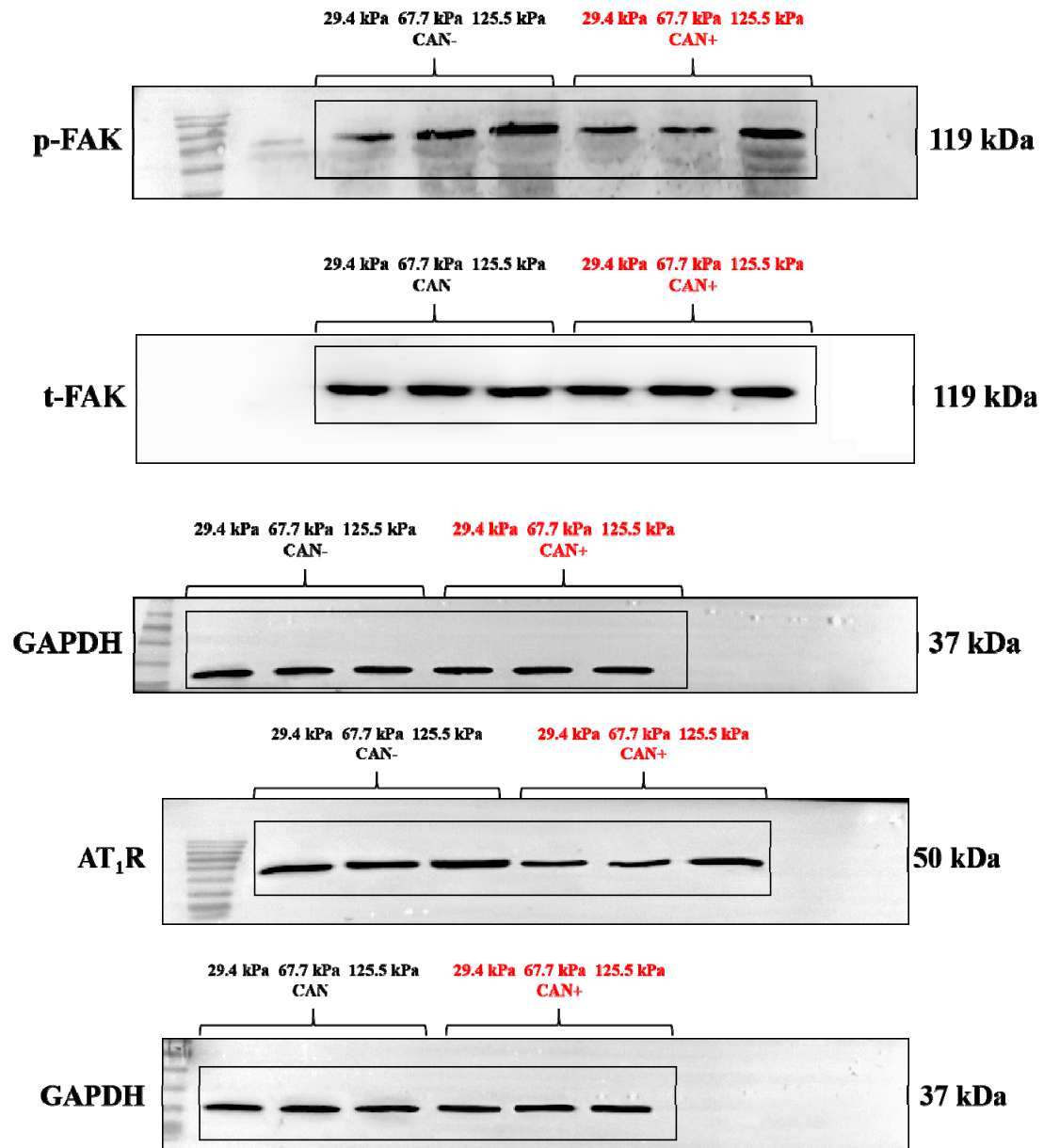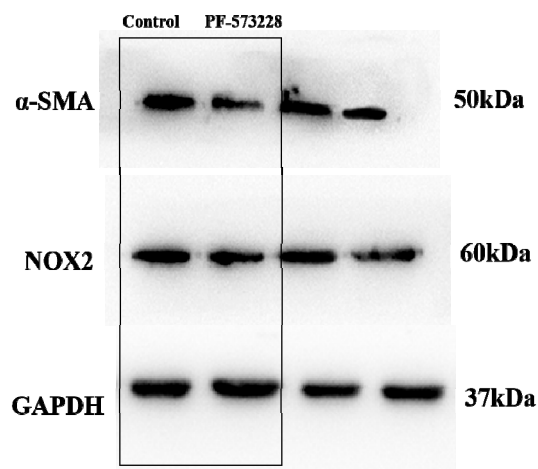

Supplement: Supplementary file 1 [file antioxidants-12-00679-s001.zip › Original Images of Western blotting.pdf]
